# An Artificial Neural Network Stratifies the Risks of Reintervention and Mortality after Endovascular Aneurysm Repair; a Retrospective Observational study

**DOI:** 10.1371/journal.pone.0129024

**Published:** 2015-07-15

**Authors:** Alan Karthikesalingam, Omneya Attallah, Xianghong Ma, Sandeep Singh Bahia, Luke Thompson, Alberto Vidal-Diez, Edward C. Choke, Matt J. Bown, Robert D. Sayers, Matt M. Thompson, Peter J. Holt

**Affiliations:** 1 Department of Outcomes Research, St George’s Vascular Institute, London, SW17 0QT, United Kingdom; 2 Vascular Surgery Group, University of Leicester, Robert Kilpatrick Clinical Sciences Building, Leicester Royal Infirmary, Leicester, LE2 7LX, United Kingdom; 3 Department of Community Health Sciences, St George’s University of London, London, SW17 0QT, United Kingdom; 4 College of Engineering and Applied Science, Aston University, Birmingham, B4 7ET, United Kingdom; 5 Department of Electronics and Communications Engineering, Arab Academy for Science and Technology and Maritime Transport, Alexandria, Egypt; Medical University Innsbruck, AUSTRIA

## Abstract

**Background:**

Lifelong surveillance after endovascular repair (EVAR) of abdominal aortic aneurysms (AAA) is considered mandatory to detect potentially life-threatening endograft complications. A minority of patients require reintervention but cannot be predictively identified by existing methods. This study aimed to improve the prediction of endograft complications and mortality, through the application of machine-learning techniques.

**Methods:**

Patients undergoing EVAR at 2 centres were studied from 2004-2010. Pre-operative aneurysm morphology was quantified and endograft complications were recorded up to 5 years following surgery. An artificial neural networks (ANN) approach was used to predict whether patients would be at low- or high-risk of endograft complications (aortic/limb) or mortality. Centre 1 data were used for training and centre 2 data for validation. ANN performance was assessed by Kaplan-Meier analysis to compare the incidence of aortic complications, limb complications, and mortality; in patients predicted to be low-risk, versus those predicted to be high-risk.

**Results:**

761 patients aged 75 +/- 7 years underwent EVAR. Mean follow-up was 36+/- 20 months. An ANN was created from morphological features including angulation/length/areas/diameters/volume/tortuosity of the aneurysm neck/sac/iliac segments. ANN models predicted endograft complications and mortality with excellent discrimination between a low-risk and high-risk group. In external validation, the 5-year rates of freedom from aortic complications, limb complications and mortality were 95.9% vs 67.9%; 99.3% vs 92.0%; and 87.9% vs 79.3% respectively (p<0.001)

**Conclusion:**

This study presents ANN models that stratify the 5-year risk of endograft complications or mortality using routinely available pre-operative data.

## Introduction

Endovascular aneurysm repair (EVAR) is the most frequently employed treatment for patients with large abdominal aortic aneurysms (AAA). The key challenge for EVAR is to ensure the durability of repair and detect endograft-related aortic complications requiring reintervention. These complications comprise a group of well-defined entities, each of which predispose to aneurysm rupture if left untreated (type 1 or 3 endoleak, sac expansion, or device migration). Limb complications (stenosis or occlusion) do not predispose to rupture but are frequently also a cause for re-intervention. Clinical trials have suggested that aortic or limb complications may affect up to 1 in 5 patients in the first 5 years after EVAR[1–4].

In order to detect these complications before aortic rupture, lifelong surveillance imaging is currently considered mandatory[5]. There is widespread debate regarding optimal surveillance intervals and the preferred imaging modality employed in endograft surveillance[6]. Despite the importance of detecting endograft related complications, only 30–50% of patients are compliant with aortic surveillance after EVAR[7]. In addition, a number of studies have found that the majority of aortic complications develop in the interval between normal surveillance scans[8–11]. The majority of endograft complications are not identified by surveillance, and over 90% of re-interventions after EVAR are prompted by the onset of symptoms between apparently normal surveillance scans[1, 9–11]. Therefore, it can be argued that over 90% of patients do not directly benefit from surveillance imaging, but remain exposed to unnecessary nephrotoxic contrast[12] and radiation[13], and incur economic cost[14]. Consequently, defining the role for endograft surveillance has been identified as a priority[15, 16], particularly in light of the significant contribution of surveillance to the cost-effectiveness of EVAR[15]. The need for surveillance is predicated entirely on the risk of endograft complications, highlighting the need for a clinical tool to predict these events.

The risk of endograft-related complications is heterogeneous[8] and a minority of patients fall within a high-risk cohort[8]. Endograft complications and reinterventions are related to pre-operative aneurysm morphology[17, 18], but current statistical models have proved insufficiently discriminatory for clinical use. Furthermore, existing risk models have not proved capable of predicting limb complications such as stenosis or occlusion, which remain a source of morbidity.

It is plausible that by using advanced machine learning techniques, such as artificial neural networks (ANNs), the prediction of endograft complications and long-term mortality after EVAR might be improved. Machine learning techniques have the potential to exploit complex and subtle relationships between pre-operative variables in order to predict the risk of post-operative events, and have gained popularity for modelling long-term outcomes in a variety of surgical settings[19–23].

The aim of the present study was to use ANN analysis to develop and externally validate a predictive tool for determining the risk of endograft complications and mortality after EVAR.

## Methods

Prospective databases were maintained for 761 patients undergoing EVAR at 2 regional vascular units in the UK, from 2004 to 2010. Details of this cohort have been published previously[17]. Data from both centres were combined to create a single database. The dataset contained details of pre-operative demographics, comorbidity and aortic morphology; peri-operative technical details including operative procedure, endograft configuration and operative adjuncts; and follow-up information including aortic complications or mortality.

The primary outcome measure was the development of endograft complications. Endograft complications were classified as aortic or limb. Aortic complications were defined on an intention-to-treat basis as a group of conditions comprising any of: aortic rupture, type 1 endoleak, type 2 endoleak with sac expansion > 5mm on CT, type 3 endoleak, sac expansion of any cause > 5mm on CT and graft migration > 5mm on CT. Limb complications were defined on an intention-to-treat basis as limb stenosis requiring reintervention; or occlusion. Stenosis was treated symptomatically and the minimal criterion for haemodynamic significance was defined as a 2.5-fold increase in peak systolic velocity on duplex ultrasound[24]. The secondary outcome measure was all-cause mortality.

### Inclusion Criteria

The study included all cases of EVAR for non-ruptured infrarenal AAA (both elective and non-elective admissions) presenting between January 2004 and June 2010 at St George’s Vascular Institute (centre 1) and Leicester Vascular Unit (centre 2). Patients undergoing open aneurysm repair, fenestrated or branch stent-grafts, or those with juxta-renal/supra-renal/thoraco-abdominal aneurysms were excluded. All data was analysed retrospectively and was anonymised and de-identified prior to analysis.

### Completion Imaging, Endograft Surveillance and Reintervention Policy

Biplanar angiography was performed at the completion of EVAR. In both centres, the role of surveillance CT imaging changed during the course of the study, although the role of duplex ultrasound remained constant. Before September 2007, postoperative imaging comprised CT and duplex prior to discharge from hospital. This group of patients underwent contrast-enhanced CT at 3 months and 1 year postoperatively. Following September 2007, follow-up comprised duplex ultrasound only, with patients undergoing duplex ultrasound and plain abdominal radiograph prior to discharge from hospital. Throughout the study, all patients underwent duplex ultrasound and plain radiography at 6 weeks, 3 months, 6 months, 9 months, 12 months, 18 months and annually thereafter.

In all cases where surveillance detected a clinically significant complication, or when patients presented symptomatically between surveillance scans, a contrast-enhanced CT was performed to direct reintervention. All patients underwent clinical evaluation at 6 weeks and 12 months after the index procedure and annually thereafter. An aggressive reintervention policy was followed for type 1 endoleak, type 2 endoleak with sac expansion > 5mm on CT, type 3 endoleak, and graft migration > 5mm.

### Data Collection: Comorbidity, Morphology and Freedom from Endograft Complications

3D morphological assessment of preoperative imaging was performed on 3Mensio Vascular software (3surgery; 3Mensio Medical Imaging B.V., Bilthoven, The Netherlands), following a validated and published protocol[25]. The minimum CT slice thickness used for the 3D reconstructions was 2.5mm. Comorbidity was categorised in binary variables specified by the Royal College of Surgeons’ Charlson index for administrative data[26].

Freedom from endograft complications was reported using Kaplan-Meier analysis in both centres. Patients undergoing EVAR at centre 1 (St George’s Vascular Institute) were treated as a “model development” cohort and used exclusively for network training. Patients at centre 2 (Leicester Vascular Unit) were treated as a “model validation” cohort, and used as an independent data source, to test the predictive accuracy of the network trained using data from centre 1 only.

Validation was performed by plotting the Kaplan-Meier freedom from endograft complications in patients predicted to be at high-risk versus those predicted to be at low-risk, with comparison by the log-rank test.

### Construction of Bayesian ANNs

The censoring time of patients treated at centre 1 was used to classify patients into three groups, which developed an endograft complication within five years (high risk group), completed five years of observation without a recorded endograft complication (low risk group), or died within 5 years without a recorded endograft complication (unknown risk). Low- and high-risk groups for endograft complication were used to build two separate Bayesian networks called *B* ^*low*^ and *B* ^*high*^ respectively after applying a standard upsampling technique to balance the datasets[27]. Each censored event was compared with the inherent distribution of the high-risk group *p* ^*high*^ and inherent distribution of low risk group *p* ^*low*^, by calculating the likelihood that the event was sampled from either model. Equations used to derive these likelihoods and specify the ANN are detailed in the accompanying appendix, (Appendix A in S1 File).

A chi-square test feature selection method was applied to select 19 morphological features for ANN construction for endograft complications; conventional univariate analyses were therefore not performed for feature selection and model inclusion[28]. Three-layers back-propagation ANNs were employed to predict aortic complications for centre 2, comprising 19 input, 4 hidden and 1 output neurons respectively. Full descriptive details of the ANN structure and the ANN dependency graph are provided in Appendix B in S1 File. Clinical data regarding patients’ comorbidity were added as inputs for an ANN to predict all-cause mortality at 5 years.

Validation of ANN performance was performed by plotting the Kaplan-Meier freedom from aortic complications in patients predicted to be at high-risk versus those predicted to be at low-risk, with comparison of actual outcomes in these predicted groups by the log-rank test.

## Results

Between 1/1/2004 and 30/6/2010, 761 patients underwent EVAR of non-ruptured infrarenal AAA and had complete morphological data available for analysis. Median follow-up was 36 months (range 11–94). 89% were male, and median (interquartile range, IQR) age was 75 (70–80) years. The development cohort (centre 1, n = 475) and the validation cohort (centre 2, n = 286) had similar demographics/comorbidity and similar pre-operative aneurysm morphology (Table 1 and Table 2).

**Table 1:** Baseline Demographics and Comorbidity in Development and Validation cohorts. Univariate analysis performed with Chi-Squared or Fisher’s exact test for dichotomous data, Mann-Whitney U-test for non-normally distributed continuous data and student’s t-test for normally distributed continuous data.

Patient Demographics or Comorbidity	Centre 1 N = 475	Centre 2 N = 286	Total N = 761	p-value
Age (median)	76.00	75.00	75.00	0.239
Age (IQR)	(70.0–80.0)	(70.0–79.0)	(70.0–80.0)	
Male Gender	416/471	260/286	676/757	0.278
Male Gender (%)	(88.3%)	(90.9%)	(89.3%)	
Estimated Glomerular Filtration Rate (ml/min/1.73m^2^) (median)	70.00	66.30	68.65	0.044
Estimate GFR (ml/min/1.73m^2^) (IQR)	(53.4–84.2)	(51.8–79.6)	(52.6–82.4)	
Ischaemic Heart Disease^*^	240/475	114/248	354/723	0.273
Ischaemic Heart Disease (%)	(50.5%)	(46.0%)	(49.0%)	
Chronic Obstructive Pulmonary Disease^†^	174/475	14/286	188/761	< 0.001
Chronic Obstructive Pulmonary Disease (%)	(36.6%)	(4.9%)	(24.7%)	
Hypertension^‡^	332/475	185/259	517/734	0.673
Hypertension (%)	(69.9%)	(71.4%)	(70.4%)	
Hypercholesterolaemia^§^	337/475	140/177	477/652	0.037
Hypercholesterolaemia (%)	(70.9%)	(79.1%)	(73.2%)	
Current Smoking	180/475	47/252	227/727	< 0.001
Current Smoking (%)	(37.9%)	(18.7%)	(31.2%)	
Diabetes^||^	70/472	39/253	109/725	0.828
Diabetes (%)	(14.8%)	(15.4%)	(15.0%)	
Top Endograft Diameter (median)	28.00	28.00	28.00	0.920
Top Endograft Diameter (IQR)	(26.0–30.0)	(26.0–32.0)	(26.0–31.0)	
AortoUniIliac Device	61/637	3/63	64/700	0.257
AortoUnIliac Device (%)	(9.6%)	(4.8%)	(9.1%)	

^*^History of angina; and history or ECG evidence of previous myocardial infarction

^†^Receiving treatment for COPD, previously diagnosed with COPD, FEV1<80% predicted or FEV1/FVC<0.7

^‡^Receiving treatment or dietary advice for hypertension or recorded blood pressure of 140/90mmHg or greater on at least two occasions

^§^Receiving treatment or dietary advice for hypercholesterolaemia

^||^Receiving treatment or dietary advice for type I or type II diabetes mellitus

**Table 2:** Morphological Features included in 19-feature Neural Networks.

Feature of AAA Morphology (pre-operative)	Centre 1 N = 475	Centre 2 N = 286	Total N = 761	Ranking Importance in ANN
Maximum AAA Diameter (Median)	62.90	62.15	62.60	1
Maximum AAA Diameter (IQR)	(58.7–71.0)	(58.1–70.8)	(58.4–70.9)	
Maximum AAA Area (Median)	2917.0	2843.0	2876.5	2
Maximum AAA Area (IQR)	(2565.5–3759.5)	(2483.0–3651.0)	(2544.0–3721.0)	
AAA Sac Volume (Median)	189.70	182.35	188.75	3
AAA Sac Volume (IQR)	(147.4–276.1)	(147.4–248.1)	(147.4–263.9)	
Largest Common Iliac Diameter, measured 10mm proximal to Internal Iliac Ostium (Median)	18.70	19.70	19.10	4
Largest Common Iliac Diameter, measured 10mm proximal to Internal Iliac Ostium (IQR)	(16.0–22.6)	(17.3–23.3)	(16.4–23.0)	
Largest Common Iliac Diameter, measured 5mm proximal to Internal Iliac Ostium (Median)	18.30	19.50	18.60	5
Largest Common Iliac Diameter, measured 5mm proximal to Internal Iliac Ostium (IQR)	(15.7–21.6)	(17.0–22.6)	(16.1–22.2)	
Largest Common Iliac Diameter, measured 1mm proximal to Internal Iliac Ostium (Median)	17.55	18.80	18.00	6
Largest Common Iliac Diameter, measured 1mm proximal to Internal Iliac Ostium (IQR)	(15.3–20.4)	(16.8–21.6)	(15.8–21.0)	
Aneurysm Neck Diameter, measured at widest point (Median)	26.30	26.80	26.45	7
Aneurysm Neck Diameter, measured at widest point (IQR)	(24.2–29.8)	(24.3–29.3)	(24.3–29.6)	
Aneurysm Neck Diameter, measured 15mm below lowest renal artery (Median)	25.70	27.00	26.00	8
Aneurysm Neck Diameter, measured 15mm below lowest renal artery (IQR)	(23.3–29.1)	(24.1–29.7)	(23.7–29.4)	
Largest Iliac Tortuosity Index (Median)	1.48	1.44	1.46	9
Largest Iliac Tortuosity Index (IQR)	(1.3–1.7)	(1.3–1.6)	(1.3–1.6)	
Aneurysm Thrombus Volume (Median)	90.50	90.05	90.50	10
Aneurysm Thrombus Volume (IQR)	(55.5–141.5)	(54.9–131.2)	(55.1–138.5)	
Aneurysm Tortuosity Index (Median)	1.11	1.09	1.11	11
Aneurysm Tortuosity Index (IQR)	(1.1–1.2)	(1.1–1.1)	(1.1–1.2)	
Aneurysm Length (Median)	101.30	107.20	103.40	12
Aneurysm Length (IQR)	(88.3–117.2)	(93.2–119.5)	(89.5–118.5)	
Largest Common Iliac Volume (Median)	14.80	15.10	14.90	13
Largest Common Iliac Volume (IQR)	(10.6–23.1)	(10.9–23.1)	(10.8–23.1)	
Aneurysm Neck Diameter, measured 10mm below lowest renal artery (Median)	25.20	25.90	25.40	14
Aneurysm Neck Diameter, measured 10mm below lowest renal artery (IQR)	(22.5–28.1)	(23.6–28.7)	(23.1–28.2)	
Aneurysm Neck Angulation (Median)	30.00	48.20	37.90	15
Aneurysm Neck Angulation (IQR)	(15.8–49.5)	(35.4–64.3)	(23.0–56.3)	
Largest Common Iliac Thrombus Volume (Median)	4.90	5.80	5.20	16
Largest Common Iliac Thrombus Volume (IQR)	(3.3–7.6)	(4.2–8.8)	(3.6–8.2)	
Aneurysm Neck Tortuosity Index (Median)	1.08	1.08	1.08	17
Aneurysm Neck Tortuosity Index (IQR)	(1.08–1.09)	(1.08–1.09)	(1.08–1.09)	
Aneurysm Neck Diameter, measured 5mm below lowest renal artery (Median)	24.50	25.60	24.90	18
Aneurysm Neck Diameter, measured 5mm below lowest renal artery (IQR)	(22.3–27.2)	(23.2–27.9)	(22.5–27.5)	
Aneurysm Neck Area, measured at widest point (Median)	571.00	515.00	550.50	19
Aneurysm Neck Area, measured at widest point (IQR)	(453.0–723.0)	(429.0–613.0)	(444.0–679.0)	

**Fig 1:**
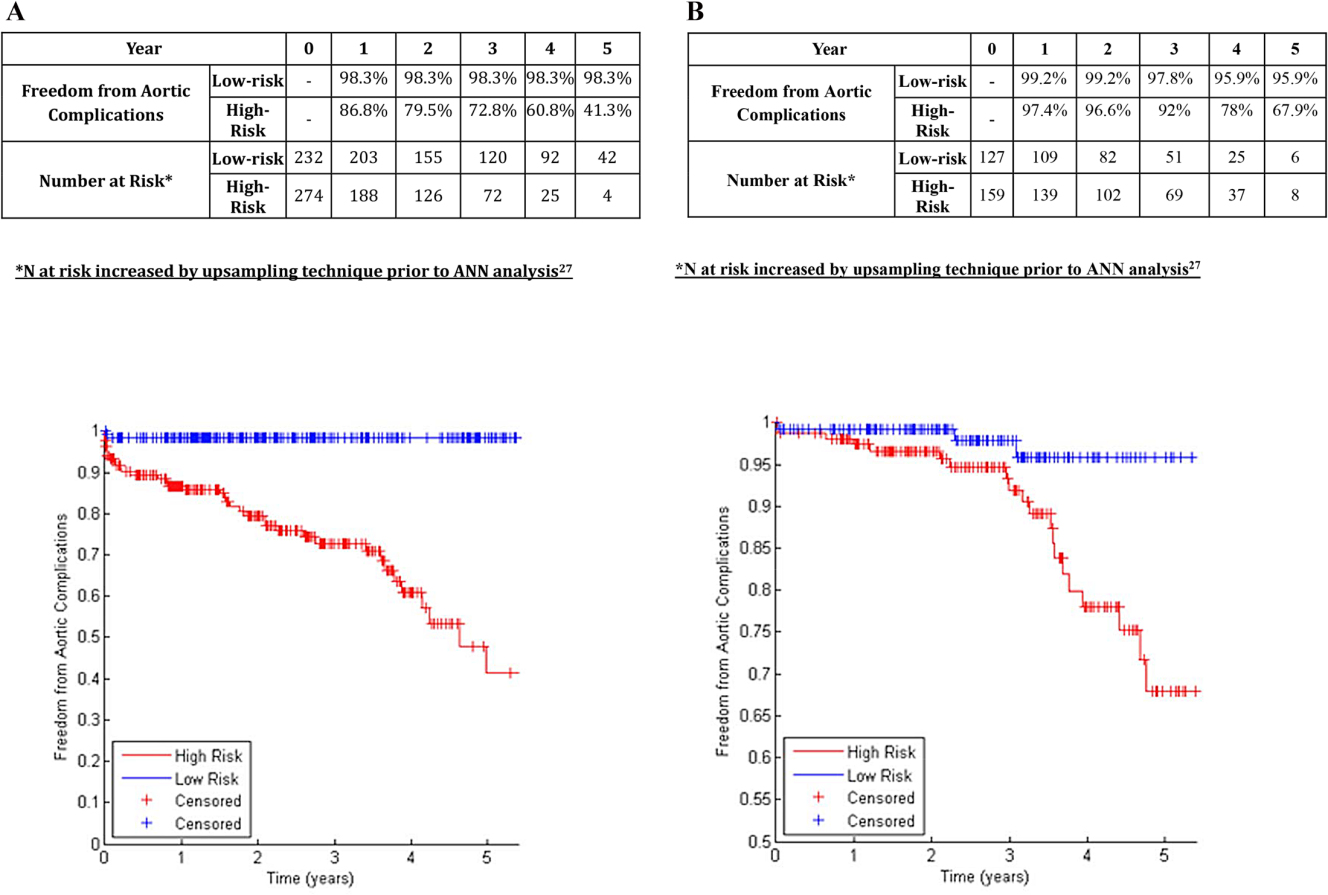
A: Freedom from Aortic Complications in Centre 1 in patients classified at low-risk or high-risk (Development Dataset). B: Freedom from Aortic Complications in Centre 2 in patients classified at low-risk or high-risk (Validation Dataset).

**Fig 2:**
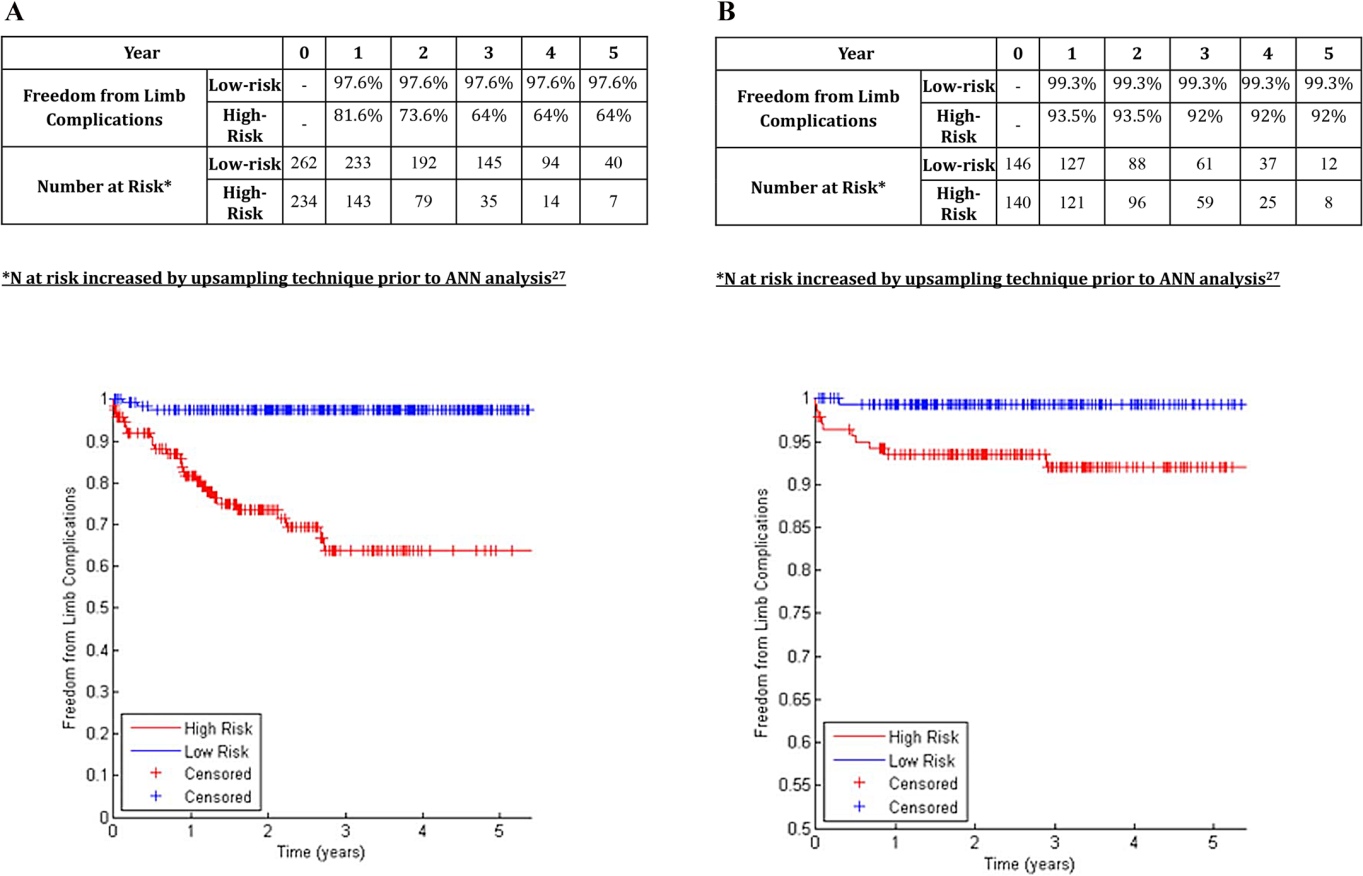
A: Freedom from Endograft Limb Complications in Centre 1 in patients classified at low-risk or high-risk (Development Dataset). B: Freedom from Endograft Limb Complications in Centre 2 in patients classified at low-risk or high-risk (Validation Dataset).

**Fig 3:**
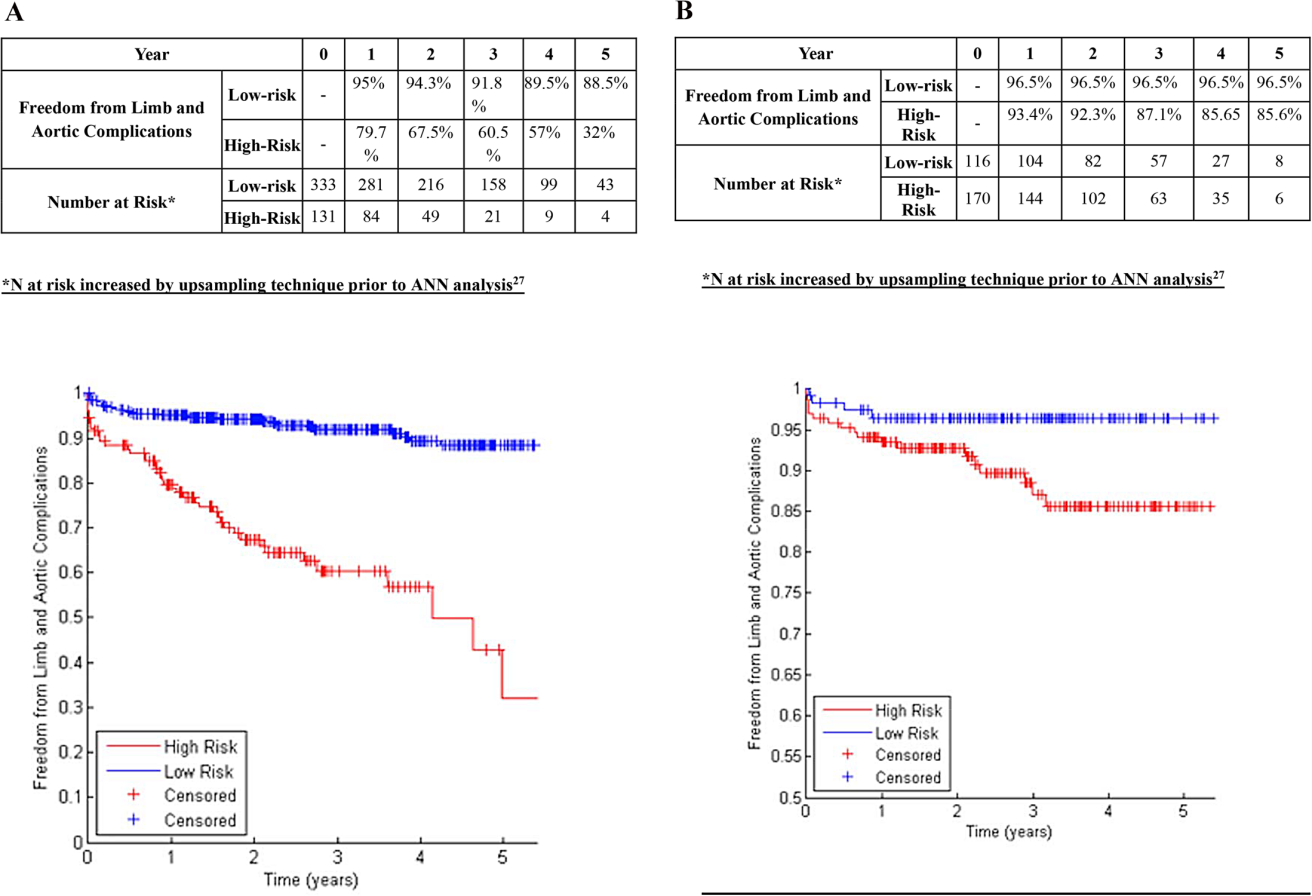
A: Freedom from All Endograft Complications in Centre 1 in patients classified at low-risk or high-risk (Development Dataset). B: Freedom from All Endograft Complications in Centre 2 in patients classified at low-risk or high-risk (Validation Dataset).

**Fig 4:**
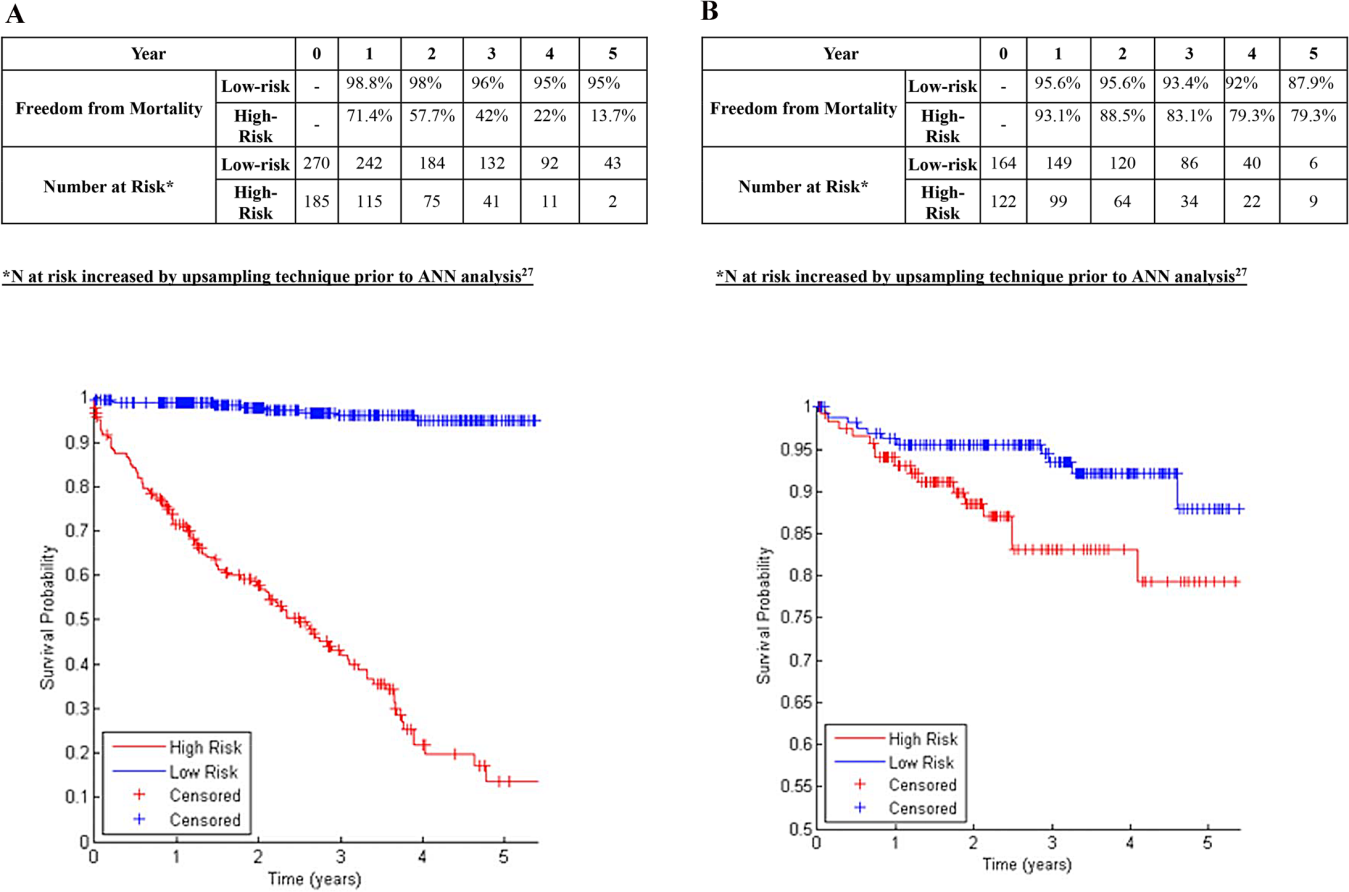
A: Freedom from Mortality in Centre 1 in patients classified at low-risk or high-risk (Development Dataset). B: Freedom from Mortality in Centre 2 in patients classified at low-risk or high-risk (Validation Dataset).

A chi-squared filter feature selection method ranked the importance of various aspects of aortic morphology as shown in Table 2, with higher-ranking features demonstrating greater predictive power for determining the risk of endograft complications. The 19 highest-ranking features were included in ANNs to predict aortic complications and limb complications (Table 2, Figs 1, 2 and 3). Binary data regarding patient comorbidity and demographics (Table 1) were added as inputs for an ANN to predict mortality (Fig 4).

### Prediction of Endograft Aortic Complications

In the training dataset (centre 1), the 19-feature Bayesian ANN allocated 45.8% of patients to the low-risk group. The 5-year freedom from aortic complications was 98.3% in the low-risk group, and 41.3% in the high-risk group (p<0.001, log-rank test; Fig 1A; c-statistic 0.763). In the validation dataset (centre 2), the ANN allocated 44.4% of patients to the low-risk group. The 5-year freedom from aortic complications was 95.9% in the low-risk group, and 67.9% in the high-risk group (p<0.001, log-rank test; Fig 1B; c-statistic 0.759).

### Prediction of Endograft Limb Complications

In the training dataset (centre 1), the 19-feature Bayesian ANN allocated 52.8% of patients to the low-risk group. The 5-year freedom from endograft limb complications was 97.6% in the low-risk group, and 64.0% in the high-risk group (p<0.001, log-rank test; Fig 2A; c-statistic 0.834). In the validation dataset (centre 2), the ANN allocated 51.0% of patients to the low-risk group. The 5-year freedom from endograft limb complications was 99.3% in the low-risk group, and 92% in the high-risk group (p<0.001, log-rank test; Fig 2B; c-statistic 0.767).

### Combined Prediction of All Endograft Complications

In the training dataset (centre 1), the 19-feature Bayesian ANN allocated 71.8% of patients to the low-risk group. The 5-year freedom from all endograft complications was 88.5% in the low-risk group, and 32.0% in the high-risk group (p<0.001, log-rank test; Fig 3A; c-statistic 0.779). In the validation dataset (centre 2), the ANN allocated 40.6% of patients to the low-risk group. The 5-year freedom from endograft limb complications was 96.5% in the low-risk group, and 85.6% in the high-risk group (p<0.001, log-rank test; Fig 3B; c-statistic 0.776).

### Prediction of Mortality after EVAR

An ANN combining 19 morphological features (Table 2) with patient demographics and comorbidity (Table 1) classified 59.3% of the training set patients (Centre 1) as low-risk. In the training dataset, the 5-year freedom from mortality was 95% in patients predicted to be low-risk, and 13.7% in those predicted to be high-risk (p<0.001 log rank test; Fig 4A; c-statistic 0.741). In the validation dataset, 57.3% were classified as low-risk and the 5-year freedom from mortality was 87.9% in the low-risk group vs. 79.3% in the high-risk group (p<0.001 log-rank test; Fig 4B; c-statistic 0.699).

## Discussion

Although the short-term benefit of EVAR for AAA is clear, challenges remain regarding its longer-term clinical success, especially endograft-related complications and all-cause mortality[2–4]. The present study demonstrated that these long-term outcomes could be risk-stratified before surgery, using routinely available pre-operative data. A machine-learning technique reproducibly categorised patients’ 5-year risk for both endograft complications and all-cause mortality. This adds considerably to previously published work aimed at providing patients with individualised risk profiles (IRP) for EVAR[17, 29]. Risk stratification of endograft complications and mortality has considerable relevance because the need for lifelong surveillance after EVAR is predicated entirely on the incidence of endograft complications, while better prediction of long-term mortality informs patient selection for surgery. All-cause mortality was reported preferentially to aneurysm-related mortality, in order to maximise internal validity and the reproducibility of our findings. This is because autopsy is rare in the UK and aneurysm-related mortality may be confounded by differences in endograft surveillance practice, whereas all-cause mortality and endograft reinterventions can be reported with confidence, and reproducibly studied. Patients identified by an ANN to be at very high risk of all-cause mortality might also be potentially excluded from consideration for AAA repair.

The ANN models developed in this study outperformed a validated IRP, the SGVI (St George’s Vascular Institute) score, which was derived using Cox Proportional Hazards modelling techniques[17] (see Appendix C Figs 1, 2, 3 and 4 in S1 File). The majority of patients are low-risk, both in clinical practice and in classification by ANN or SGVI score models. The ANN provided a more powerful prediction of the low-risk group than has previously been possible with the SGVI score, which resulted in superior performance in simulated surveillance studies (see Appendix D in S1 File). This finding is not unexpected due to the construct of ANNs; that enables them to outperform logistic or survival models by including multiple interacting and complex covariate effects for event prediction. To date, ANNs have successfully been used in the clinical environment as event predictors in a variety of settings including the diagnosis of myocardial infarction[30] and survival after acute coronary syndrome[31]. In other clinical arenas, studies have demonstrated that ANNs outperform logistic regression models in the prediction of autonomic dysfunction[32], or mortality following hip fracture[33]. These quoted studies compared ANN with models derived from logistic regression; the SGVI score was derived using Cox Proportional Hazards modelling techniques and similar comparisons in this context have also been previously carried out[34].

The impact of aortic morphology on long-term outcome of EVAR is complex and well-suited to ANN analysis, with considerable potential for interaction between aortic volume, shape, diameter, angulation. Existing models have repeatedly demonstrated that aneurysm diameter predicts reintervention after EVAR[35, 36], but evidence also suggests that other aspects of aneurysm morphology contribute to long-term clinical success[23, 37–39]. More complex considerations such as endograft configuration and deployment, or intermediate markers of patients’ cardiovascular risk phenotype, could potentially be “learned” by future iterations of the ANN in prospective studies. The addition of further operative factors (length of operation, graft size, and endoleak at completion) or post-operative factors (endoleak at early surveillance scans) might further improve the discriminatory power of ANNs.

In contrast to conventional statistical models, the ANN utilised for the present study was designed as a binary classifier of a dichotomous event: endograft complications, or all-cause mortality. A limitation of this technique is that clinicians are not provided with a greater number of predicted risk strata, or a group at predicted intermediate risk. A multinomial modification of the ANN analysis technique can allow for generation of intermediate risk strata, but there were too few patients to allow adequate discrimination in this study (Appendix E in S1 File); while for endograft surveillance a dichotomous model arguably provides sufficient information for clinicians to identify those in whom surveillance could either be curtailed or intensified. A disadvantage of the ANN approach was that the best model required the measurement and input of 19 features of aortic morphology for maximum accuracy, compromising ease of use for clinicians. A more parsimonious ANN, incorporating 8 predictive features, would allow faster use by clinicians, but performed with inferior discriminatory power (see Appendix C Figs 1, 2, 3 and 4 in S1 File). A web portal for entry of morphology data, relaying information to an ANN hosted on a remote server, would mitigate this difficulty and improve the usability of the ANN solution for clinicians. A single software package was used for the assessment of aortic morphology in the present study to minimise bias and maximise reproducibility. However, for clinical practice, any locally available software allowing 3D reconstruction of CT aortograms would be suitable, further improving the acceptability of this solution. Furthermore, the model was developed and validated on a cohort of patients from two institutions in a single country. It was notable that the proportion of patients classified as high- or low-risk varied between centres, although the absolute difference in event rates between groups within each centre was clinically significant. This might be attributable to local differences in patients’ preferences for endograft limb reinterventions, and further prospective evaluation of the ANN models will be essential to inform clinicians regarding its generalizability, and improve understanding of the potential health economic implications for stratified endograft surveillance. A further potential limitation of the ANN technique was the number of input variables required compared to the number of events detected; this might challenge the generalizability of the model and further evidence is required of its performance characteristics in other groups of patients.

It has previously been suggested that surveillance should be adjusted according to patients’ AAA diameter as a surrogate for the future risk of endograft complication[35]. Unfortunately, existing IRPs have not been able to define the lowest-risk patients with sufficient accuracy to suggest safe termination of surveillance in such a cohort; for example, the lowest-risk patients classified by the SGVI score continued to demonstrate a 12% 5-year risk of aortic complications[17]. Furthermore, existing risk scores have not been able to predict endograft limb complications (stenosis or occlusion), which remain a source of morbidity[24].

The ANN models developed in the present study have the potential to inform surveillance practice by stratifying the incidence of all endograft complications requiring reintervention. Existing controversies around surveillance after EVAR encompass diagnostic inaccuracy, patient attendance rates, interval presentations and adverse events caused through surveillance including ultimately unrequired reinterventions, nephrotoxicity, radiation exposure and cost. Due to these issues, surveillance protocols tend to be applied uniformly, and are empirical rather than evidence-based. There is widespread variation in practice in the UK[40, 41] and internationally[42], both in the timing of scans and the imaging modality used. Many centres utilise CT for first-line imaging amid concerns regarding the sensitivity of duplex, but duplex ultrasound is proven to offer sufficient diagnostic accuracy for detecting key complications[6], is safe [43, 44] and is preferred to CT by institutions with greater experience in performing EVAR[42].

Several studies have reported that the majority of complications requiring reintervention after EVAR are most often detected by the onset of symptoms occurring in the interval between apparently normal surveillance scans[1, 9–11]; so that just 1.4–9% of patients undergo reintervention after EVAR directly as a result of surveillance findings rather than the development of symptoms between surveillance scans. Therefore, it could be argued that over 90% of all patients receive no benefit from post-EVAR surveillance. In addition, 15% of post-EVAR aneurysm ruptures are described in patients with no detectable stent abnormality on surveillance imaging[45]. Finally, the attendance rate at surveillance is globally less than 50% of those who originally underwent EVAR, with some reports suggesting that the figure is as low as 30%[7, 46]. Surveillance can also give rise to false positive findings; in one series, 3/553 (0.5%) patients underwent unnecessary diagnostic angiography due to apparent endoleak on initial surveillance; exposing these patients to unnecessary procedural risk[8]. The present study suggests that an ANN technique for analysing aortic morphology and basic co-morbidity data might inform a more evidence-based approach to patient selection and post-operative surveillance, in which the frequency of scans can be targeted to the risk of endograft failure.

## Conclusion

This study has demonstrated that it is possible to stratify the risk of key long-term outcomes after EVAR, based on routinely available pre-operative data combined with accurate assessment of aortic morphology. This might impact both patient selection and surveillance after EVAR, which remain subject to controversy in practice. The development of a user interface, further validation of the model and feasibility study are required to enhancing the acceptability of this proposal.

## Supporting Information

S1 File Appendices. Equations for ANN Specification, Description of ANN structure, Comparison of 19-feature ANN performance with the SGVI Score and a more parsimonious 8-feature ANN, Simulated Surveillance Protocols and Performance of a 3-group ANN for classification of high-risk, medium-risk, and low-risk patients for limb or aortic endograft complications after EVAR.
(DOC)

## References

[1] Nordon IM, Karthikesalingam A, Hinchliffe RJ, Holt PJ, Loftus IM, Thompson MM. Secondary interventions following endovascular aneurysm repair (EVAR) and the enduring value of graft surveillance. European journal of vascular and endovascular surgery: the official journal of the European Society for Vascular Surgery. 2010 5;39(5):547–54. . Epub 2009/11/27. eng. 19939711 10.1016/j.ejvs.2009.11.002

[2] Lederle FA, Freischlag JA, Kyriakides TC, Matsumura JS, Padberg FT Jr, Kohler TR, et al Long-term comparison of endovascular and open repair of abdominal aortic aneurysm. The New England journal of medicine. 2012 11 22;367(21):1988–97. . Epub 2012/11/23. eng. 23171095 10.1056/NEJMoa1207481

[3] De Bruin JL, Baas AF, Buth J, Prinssen M, Verhoeven EL, Cuypers PW, et al Long-term outcome of open or endovascular repair of abdominal aortic aneurysm. The New England journal of medicine. 2010 5 20;362(20):1881–9. Epub 2010/05/21. eng. 10.1056/NEJMoa0909499 20484396

[4] United Kingdom ETI, Greenhalgh RM, Brown LC, Powell JT, Thompson SG, Epstein D, et al Endovascular versus open repair of abdominal aortic aneurysm. N Engl J Med. 2010 5 20;362(20):1863–71. 10.1056/NEJMoa0909305 20382983

[5] Moll FL, Powell JT, Fraedrich G, Verzini F, Haulon S, Waltham M, et al Management of abdominal aortic aneurysms clinical practice guidelines of the European society for vascular surgery. European journal of vascular and endovascular surgery: the official journal of the European Society for Vascular Surgery. 2011 1;41 Suppl 1:S1–S58. . 21215940 10.1016/j.ejvs.2010.09.011

[6] Karthikesalingam A, Al-Jundi W, Jackson D, Boyle JR, Beard JD, Holt PJ, et al Systematic review and meta-analysis of duplex ultrasonography, contrast-enhanced ultrasonography or computed tomography for surveillance after endovascular aneurysm repair. The British journal of surgery. 2012 11;99(11):1514–23. Epub 2012/09/25. eng. 10.1002/bjs.8873 23001681

[7] Kret MR, Azarbal AF, Mitchell EL, Liem TK, Landry GJ, Moneta GL. Compliance with long-term surveillance recommendations following endovascular aneurysm repair or type B aortic dissection. Journal of vascular surgery: official publication, the Society for Vascular Surgery [and] International Society for Cardiovascular Surgery, North American Chapter. 2013 7;58(1):25–31. . 23465175 10.1016/j.jvs.2012.12.046

[8] Karthikesalingam A, Holt PJ, Hinchliffe RJ, Nordon IM, Loftus IM, Thompson MM. Risk of reintervention after endovascular aortic aneurysm repair. Br J Surg. 2010 5;97(5):657–63. 10.1002/bjs.6991 20235086

[9] Chisci E, Setacci F, Iacoponi F, de Donato G, Cappelli A, Setacci C. Surveillance imaging modality does not affect detection rate of asymptomatic secondary interventions following EVAR. European journal of vascular and endovascular surgery: the official journal of the European Society for Vascular Surgery. 2012 3;43(3):276–81. . Epub 2012/01/14. eng. 22240330 10.1016/j.ejvs.2011.11.020

[10] Dias NV, Riva L, Ivancev K, Resch T, Sonesson B, Malina M. Is there a benefit of frequent CT follow-up after EVAR? European Journal of Vascular and Endovascular Surgery. 2009;37(4):425–30. 10.1016/j.ejvs.2008.12.019 19233689

[11] Black SA, Carrell TW, Bell RE, Waltham M, Reidy J, Taylor PR. Long-term surveillance with computed tomography after endovascular aneurysm repair may not be justified. Br J Surg. 2009 11;96(11):1280–3. 10.1002/bjs.6732 19847868

[12] Walsh SR, Tang TY, Boyle JR. Renal consequences of endovascular abdominal aortic aneurysm repair. Journal of endovascular therapy: an official journal of the International Society of Endovascular Specialists. 2008 2;15(1):73–82. . 18254679 10.1583/07-2299.1

[13] Weerakkody RA, Walsh SR, Cousins C, Goldstone KE, Tang TY, Gaunt ME. Radiation exposure during endovascular aneurysm repair. Br J Surg. 2008 6;95(6):699–702. 10.1002/bjs.6229 18446782

[14] Michaels JA, Drury D, Thomas SM. Cost-effectiveness of endovascular abdominal aortic aneurysm repair. The British journal of surgery. 2005 8;92(8):960–7. . 16034841 10.1002/bjs.5119

[15] Brown L, Powell J, Thompson S, Epstein D, Sculpher M, Greenhalgh R. The UK EndoVascular Aneurysm Repair (EVAR) trials: randomised trials of EVAR versus standard therapy. Health Technol Assess. 2012 2;16(9):1–218. Epub 2012/03/03. eng. 10.3310/hta16090 22381040

[16] Chambers D, Epstein D, Walker S, Fayter D, Paton F, Wright K, et al Endovascular stents for abdominal aortic aneurysms: a systematic review and economic model. Health Technol Assess. 2009 10;13(48):1–189. Epub 2009/10/24. eng. 10.3310/hta13480 19849958

[17] Karthikesalingam A, Holt PJ, Vidal-Diez A, Choke EC, Patterson BO, Thompson LJ, et al Predicting aortic complications after endovascular aneurysm repair. The British journal of surgery. 2013 9;100(10):1302–11. Epub 2013/06/26. eng. 10.1002/bjs.9177 23797788

[18] Schanzer A, Greenberg RK, Hevelone N, Robinson WP, Eslami MH, Goldberg RJ, et al Predictors of abdominal aortic aneurysm sac enlargement after endovascular repair. Circulation. 2011 6 21;123(24):2848–55. Epub 2011/04/12. eng. 10.1161/CIRCULATIONAHA.110.014902 21478500

[19] Ansari D, Nilsson J, Andersson R, Regner S, Tingstedt B, Andersson B. Artificial neural networks predict survival from pancreatic cancer after radical surgery. American journal of surgery. 2013 1;205(1):1–7. Epub 2012/12/19. eng. 10.1016/j.amjsurg.2012.05.032 23245432

[20] Bartosch-Harlid A, Andersson B, Aho U, Nilsson J, Andersson R. Artificial neural networks in pancreatic disease. The British journal of surgery. 2008 7;95(7):817–26. Epub 2008/06/14. eng. 10.1002/bjs.6239 18551536

[21] Spelt L, Nilsson J, Andersson R, Andersson B. Artificial neural networks—a method for prediction of survival following liver resection for colorectal cancer metastases. European journal of surgical oncology: the journal of the European Society of Surgical Oncology and the British Association of Surgical Oncology. 2013 6;39(6):648–54. . Epub 2013/03/22. eng. 23514791 10.1016/j.ejso.2013.02.024

[22] Nilsson J, Ohlsson M, Thulin L, Hoglund P, Nashef SA, Brandt J. Risk factor identification and mortality prediction in cardiac surgery using artificial neural networks. The Journal of thoracic and cardiovascular surgery. 2006 7;132(1):12–9. . Epub 2006/06/27. eng. 16798296 10.1016/j.jtcvs.2005.12.055

[23] Lo BW, Macdonald RL, Baker A, Levine MA. Clinical outcome prediction in aneurysmal subarachnoid hemorrhage using Bayesian neural networks with fuzzy logic inferences. Computational and mathematical methods in medicine. 2013;2013:904860 Pubmed Central PMCID: 3639630. 10.1155/2013/904860 23690884 PMC3639630

[24] Karthikesalingam A, Kumar S, Anandarajah JJ, Hinchliffe RJ, Poloniecki JD, Thompson MM, et al Predictive value of peak systolic velocity for the development of graft limb complications after endovascular aneurysm repair. Journal of endovascular therapy: an official journal of the International Society of Endovascular Specialists. 2012 6;19(3):428–33. . Epub 2012/07/14. eng. 22788897 10.1583/11-3739MR.1

[25] Ghatwary T, Karthikesalingam A, Patterson B, Hinchliffe R, Morgan R, Loftus I, et al St George's Vascular Institute protocol: an accurate and reproducible methodology to enable comprehensive characterization of infrarenal abdominal aortic aneurysm morphology in clinical and research applications. Journal of endovascular therapy: an official journal of the International Society of Endovascular Specialists. 2012 6;19(3):400–14. . Epub 2012/07/14. eng. 22788895 10.1583/11-3731MR.1

[26] Armitage JN, van der Meulen JH. Identifying co-morbidity in surgical patients using administrative data with the Royal College of Surgeons Charlson Score. The British journal of surgery. 2010 5;97(5):772–81. Epub 2010/03/23. eng. 10.1002/bjs.6930 20306528

[27] Wei Q, Dunbrack RL Jr. The role of balanced training and testing data sets for binary classifiers in bioinformatics. PloS one. 2013;8(7):e67863 Pubmed Central PMCID: 3706434. 10.1371/journal.pone.0067863 23874456 PMC3706434

[28] Lagani V, Tsamardinos I. Structure-based variable selection for survival data. Bioinformatics. 2010 8 1;26(15):1887–94. Epub 2010/06/04. 10.1093/bioinformatics/btq261 20519286

[29] Johnson PG, Chipman CR, Ahanchi SS, Kim JH, Dexter DJ, Panneton JM. A case-matched validation study of anatomic severity grade score in predicting reinterventions after endovascular aortic aneurysm repair. Journal of vascular surgery. 2013 9;58(3):582–8. Epub 2013/06/19. eng. 10.1016/j.jvs.2013.03.045 23769938

[30] Baxt WG, Shofer FS, Sites FD, Hollander JE. A neural computational aid to the diagnosis of acute myocardial infarction. Annals of emergency medicine. 2002 4;39(4):366–73. . Epub 2002/03/29. eng. 11919522 10.1067/mem.2002.122705

[31] Harrison RF, Kennedy RL. Artificial neural network models for prediction of acute coronary syndromes using clinical data from the time of presentation. Annals of emergency medicine. 2005 11;46(5):431–9. . 16271675 10.1016/j.annemergmed.2004.09.012

[32] Tang ZH, Liu J, Zeng F, Li Z, Yu X, Zhou L. Comparison of prediction model for cardiovascular autonomic dysfunction using artificial neural network and logistic regression analysis. PloS one. 2013;8(8):e70571 Pubmed Central PMCID: 3734274. Epub 2013/08/14. eng. 10.1371/journal.pone.0070571 23940593 PMC3734274

[33] Lin CC, Ou YK, Chen SH, Liu YC, Lin J. Comparison of artificial neural network and logistic regression models for predicting mortality in elderly patients with hip fracture. Injury. 2010 8;41(8):869–73. Epub 2010/05/25. eng. 10.1016/j.injury.2010.04.023 20494353

[34] Puddu PE, Menotti A. Artificial neural networks versus proportional hazards Cox models to predict 45-year all-cause mortality in the Italian Rural Areas of the Seven Countries Study. BMC medical research methodology. 2012;12:100 Pubmed Central PMCID: 3549727. 10.1186/1471-2288-12-100 22824187 PMC3549727

[35] Peppelenbosch N, Buth J, Harris PL, van Marrewijk C, Fransen G. Diameter of abdominal aortic aneurysm and outcome of endovascular aneurysm repair: does size matter? A report from EUROSTAR. Journal of vascular surgery. 2004 2;39(2):288–97. . Epub 2004/01/27. eng. 14743127 10.1016/j.jvs.2003.09.047

[36] Brown LC, Greenhalgh RM, Powell JT, Thompson SG. Use of baseline factors to predict complications and reinterventions after endovascular repair of abdominal aortic aneurysm. The British journal of surgery. 2010 8;97(8):1207–17. Epub 2010/07/06. eng. 10.1002/bjs.7104 20602502

[37] Wyss TR, Dick F, Brown LC, Greenhalgh RM. The influence of thrombus, calcification, angulation, and tortuosity of attachment sites on the time to the first graft-related complication after endovascular aneurysm repair. Journal of vascular surgery. 2011 10;54(4):965–71. Epub 2011/07/05. eng. 10.1016/j.jvs.2011.04.007 21723072

[38] Stather PW, Wild JB, Sayers RD, Bown MJ, Choke E. Endovascular aortic aneurysm repair in patients with hostile neck anatomy. Journal of endovascular therapy: an official journal of the International Society of Endovascular Specialists. 2013 10;20(5):623–37. . Epub 2013/10/08. eng. 24093314 10.1583/13-4320MR.1

[39] Stather PW, Sayers RD, Cheah A, Wild JB, Bown MJ, Choke E. Outcomes of endovascular aneurysm repair in patients with hostile neck anatomy. European journal of vascular and endovascular surgery: the official journal of the European Society for Vascular Surgery. 2012 12;44(6):556–61. . Epub 2012/11/06. eng. 23122183 10.1016/j.ejvs.2012.10.003

[40] Karthikesalingam A, Page AA, Pettengell C, Hinchliffe RJ, Loftus IM, Thompson MM, et al Heterogeneity in surveillance after endovascular aneurysm repair in the UK. European journal of vascular and endovascular surgery: the official journal of the European Society for Vascular Surgery. 2011 11;42(5):585–90. . 21783388 10.1016/j.ejvs.2011.06.053

[41] Patel A, Edwards R, Chandramohan S. Surveillance of patients post-endovascular abdominal aortic aneurysm repair (EVAR). A web-based survey of practice in the UK. Clinical radiology. 2013 6;68(6):580–7. Epub 2013/04/02. eng. 10.1016/j.crad.2012.11.019 23541091

[42] Uthoff H, Pena C, Katzen BT, Gandhi R, West J, Benenati JF, et al Current clinical practice in postoperative endovascular aneurysm repair imaging surveillance. Journal of vascular and interventional radiology: JVIR. 2012 9;23(9):1152–9 e6. 10.1016/j.jvir.2012.06.003 22854317

[43] Gray C, Goodman P, Herron CC, Lawler LP, O'Malley MK, O'Donohoe MK, et al Use of colour duplex ultrasound as a first line surveillance tool following EVAR is associated with a reduction in cost without compromising accuracy. European journal of vascular and endovascular surgery: the official journal of the European Society for Vascular Surgery. 2012 8;44(2):145–50. . Epub 2012/06/22. eng. 22717670 10.1016/j.ejvs.2012.05.008

[44] Harrison GJ, Oshin OA, Vallabhaneni SR, Brennan JA, Fisher RK, McWilliams RG. Surveillance after EVAR based on duplex ultrasound and abdominal radiography. European journal of vascular and endovascular surgery: the official journal of the European Society for Vascular Surgery. 2011 8;42(2):187–92. . Epub 2011/05/07. eng. 21546278 10.1016/j.ejvs.2011.03.027

[45] Schlosser FJ, Gusberg RJ, Dardik A, Lin PH, Verhagen HJ, Moll FL, et al Aneurysm rupture after EVAR: can the ultimate failure be predicted? European Journal of Vascular and Endovascular Surgery. 2009;37(1):15–22. 10.1016/j.ejvs.2008.10.011 19008129

[46] Jones WB, Taylor SM, Kalbaugh CA, Joels CS, Blackhurst DW, Langan EM 3rd, et al Lost to follow-up: a potential under-appreciated limitation of endovascular aneurysm repair. Journal of vascular surgery. 2007 9;46(3):434–40; discussion 40–1. . Epub 2007/09/11. eng. 17826228 10.1016/j.jvs.2007.05.002

