# Supplementary material for: An Artificial Neural Network Stratifies the Risks of Reintervention and Mortality after Endovascular Aneurysm Repair; a Retrospective Observational study
Source: PLoS One. 2015 Jul 15;10(7):e0129024. doi: 10.1371/journal.pone.0129024 (PMC4503678; doi:10.1371/journal.pone.0129024)
Supplement: S1 File — Equations for ANN Specification, Description of ANN structure, Comparison of 19-feature ANN performance with the SGVI Score and a more parsimonious 8-feature ANN, Simulated Surveillance Protocols and Performance of a 3-group ANN for classification of high-risk, medium-risk, and low-risk patients for limb or aortic endograft complications after EVAR. (DOC) [file pone.0129024.s001.doc]

**SUPPLEMENTARY MATERIAL**

APPENDIX A: Equations for ANN Specification.

Likelihood was calculated from equations (1) and (2) by multiplying all the exact probabilities from the probability tables, following network topology [19].

Equation 1: Equation 2:

The posterior probability $P(O/x_{c})$ which is the output prediction (*O*) given that it is censored ($x_{c}$) was calculated using equations (3) and (4), then normalized to ignore the effect of probability of a censored instance $P(x_{c})$ to derive equation (5).

Equation 3:

Equation 4:

Equation 5:

A censoring correction threshold $P_{Th}$ was chosen to relocate censored events to high or low risk groups, by comparing $P(O^{high}/x_{c})$ to$P_{Th}$. Centre 1 data were uncensored for network construction.

The threshold used for re-allocating censored events to either low or high risk groups was 0.5.

APPENDIX B: Description of ANN structure.

Artificial neural networks (ANN) are popular “machine-learning” classifiers. The structure of ANN simulate biological neural networks by comprising a number of “neurons” connected by “weights” (synapses). The network contains input, hidden and output layers and can iteratively “learn” from a given dataset the different ways in which input data can lead to an output classifier. The learning process in ANN is achieved by changing the weights of the connected neurons, beginning with every input feature of the training dataset. Each neuron has an activation function used to produce an output to neurons of the next layer.

In our study, the censoring time of patients treated at centre 1 was used to classify patients into three groups, which developed an endograft complication within five years (high risk group), completed five years of observation without a recorded endograft complication (low risk group), or died within 5 years without a recorded endograft complication (unknown risk). Low- and high-risk groups for endograft complication were used to build two separate Bayesian networks called $B^{low}$and $B^{high}$respectively. Each censored event was compared with the inherent distribution of the high-risk group $p^{high}$ and inherent distribution of low risk group $p^{low}$, by calculating the likelihood that the event was sampled from either model. Equations used to derive these likelihoods and specify the ANN are detailed in “Appendix 1” above. A chi-square test feature selection method was applied to select 19 morphological features for ANN construction for endograft complications; conventional univariate analyses were therefore not performed for feature selection and model inclusion. Three-layers back-propagation ANNs were employed to predict aortic complications for centre 2, comprising 19 input, 4 hidden and 1 output neurons respectively. Clinical data regarding patients’ comorbidity were added as inputs for an ANN to predict all-cause mortality at 5 years.

Each ANN was set up as follows:

| **Complication Type** | **Aortic** | **Limb** | **Mortality** |
| --- | --- | --- | --- |
| **Number of layers** | 3 | 3 | 3 |
| **Number of input neurons** | 19 | 19 | 24 |
| **Number of hidden neurons** | 4 | 9 | 8 |
| **Number of output neurons** | 1 | 1 | 1 |
| **Activation function** | Log sigmoid, satlin | Logsigmoid, satlin | Logsigmoid, satlin |
| **Mean square error** | 0.075 | 0.075 | 0.07 |

A corresponding ANN dependency graph for our study is shown below, to illustrate the arrangement of input, hidden and output neurons, and their interlinking weights:


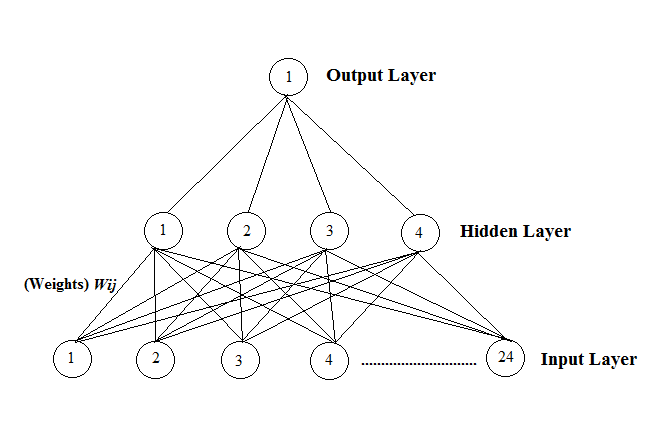


APPENDIX C: Comparison of 19-feature ANN performance with the SGVI Score and a more parsimonious 8-feature ANN

**Appendix C, Figure 1: Freedom from Aortic Complications in Centre 1 in patients classifed at low-risk or high-risk by the 19-feature neural network; compared to the SGVI Score.**

**
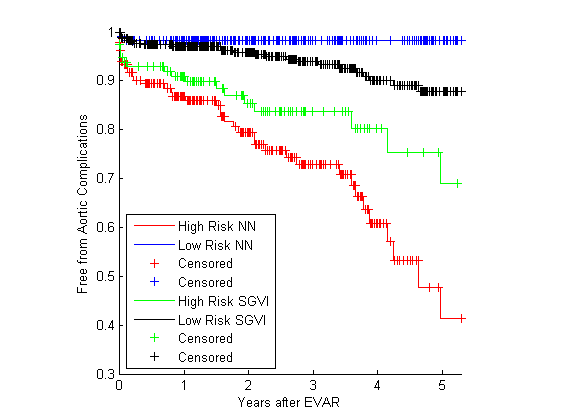
**

| **19-feature Neural Network at Year End:** | | **0** | **1** | **2** | **3** | **4** | **5** |
| --- | --- | --- | --- | --- | --- | --- | --- |
| **Freedom from Aortic Complications** | **Low-risk** | - | 98.3% | 98.3% | 98.3% | 98.3% | 98.3% |
|  | **High-Risk** | - | 86.8% | 79.5% | 72.8% | 60.8% | 41.3% |
| **Number at Risk** | **Low-risk** | 232 | 203 | 155 | 120 | 92 | 42 |
|  | **High-Risk** | 274 | 188 | 126 | 72 | 25 | 4 |
| **SGVI Score at Year End:** | | **0** | **1** | **2** | **3** | **4** | **5** |
| **Freedom from Aortic Complications** | **Low-risk** | - | 97% | 96% | 94% | 90% | 88% |
|  | **High-Risk** | - | 90% | 85% | 83% | 80% | 69% |
| **Number at Risk** | **Low-risk** | 360 | 287 | 217 | 155 | 95 | 54 |
|  | **High-Risk** | 115 | 84 | 51 | 30 | 16 | 11 |

**Appendix C, Figure 2: Freedom from Aortic Complications in Centre 2 in patients classifed at low-risk or high-risk by the 19-feature neural network; compared to the SGVI Score.**

**
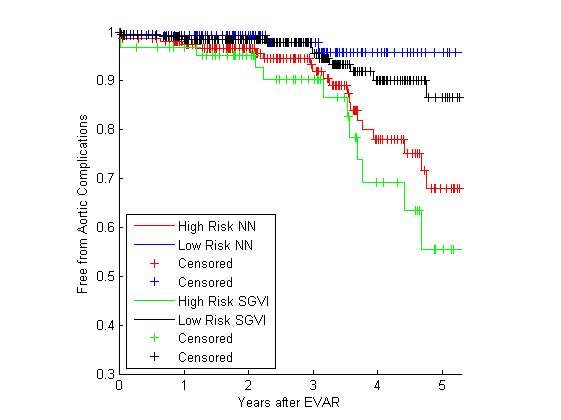
**

| **19-feature Neural Network at Year End:** | | **0** | **1** | **2** | **3** | **4** | **5** |
| --- | --- | --- | --- | --- | --- | --- | --- |
| **Freedom from Aortic Complications** | **Low-risk** | - | 99.2% | 99.2% | 97.8% | 95.9% | 95.9% |
|  | **High-Risk** | - | 97.4% | 96.6% | 92% | 78% | 67.9% |
| **Number at Risk** | **Low-risk** | 127 | 109 | 82 | 51 | 25 | 6 |
|  | **High-Risk** | 159 | 139 | 102 | 69 | 37 | 8 |
| **SGVI Score at Year End:** | | **0** | **1** | **2** | **3** | **4** | **5** |
| **Freedom from Aortic Complications** | **Low-risk** | - | 99% | 99% | 96% | 91% | 88% |
|  | **High-Risk** | - | 97% | 95% | 90% | 70% | 55% |
| **Number at Risk** | **Low-risk** | 220 | 191 | 141 | 91 | 49 | 21 |
|  | **High-Risk** | 66 | 57 | 43 | 28 | 13 | 4 |

**Appendix C, Figure 3: Freedom from Aortic Complications in Centre 1 in patients classifed at low-risk or high-risk by a more parsimonious 8-feature neural network; compared to the SGVI Score.**


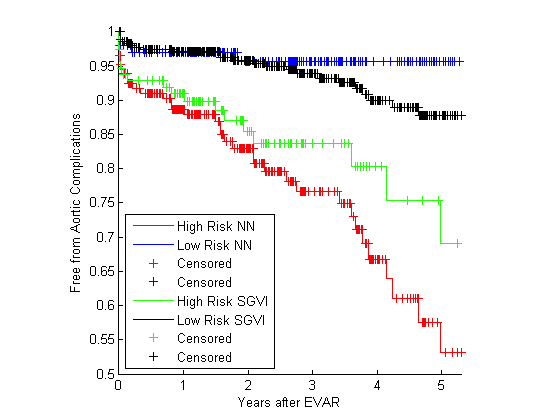


| **8-feature Neural Network at Year End:** | | **0** | **1** | **2** | **3** | **4** | **5** |
| --- | --- | --- | --- | --- | --- | --- | --- |
| **Freedom from Aortic Complications** | **Low-risk** | - | 97% | 95.62% | 95.62% | 95.62% | 95.62% |
|  | **High-Risk** | - | 88.73% | 83% | 76.72% | 66.77% | 53.17% |
| **Number at Risk** | **Low-risk** | 208 | 174 | 128 | 96 | 64 | 35 |
|  | **High-Risk** | 298 | 215 | 153 | 96 | 53 | 21 |
| **SGVI Score at Year End:** | | **0** | **1** | **2** | **3** | **4** | **5** |
| **Freedom from Aortic Complications** | **Low-risk** | - | 97% | 96% | 94% | 90% | 88% |
|  | **High-Risk** | - | 90% | 85% | 83% | 80% | 69% |
| **Number at Risk** | **Low-risk** | 360 | 287 | 217 | 155 | 95 | 54 |
|  | **High-Risk** | 115 | 84 | 51 | 30 | 16 | 11 |

**Appendix C, Figure 4: Freedom from Aortic Complications in Centre 2 in patients classifed at low-risk or high-risk by a more parsimonious 8-feature neural network; compared to the SGVI Score.**


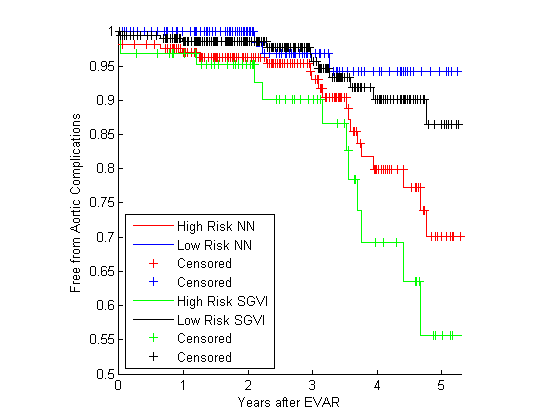


| **8-feature Neural Network at Year End:** | | **0** | **1** | **2** | **3** | **4** | **5** |
| --- | --- | --- | --- | --- | --- | --- | --- |
| **Freedom from Aortic Complications** | **Low-risk** | - | 99.9% | 99.9% | 97% | 94.2% | 94.2% |
|  | **High-Risk** | - | 97% | 96.3% | 93% | 79.8% | 70.15% |
| **Number at Risk** | **Low-risk** | 115 | 100 | 73 | 41 | 25 | 5 |
|  | **High-Risk** | 171 | 150 | 113 | 80 | 39 | 10 |
| **SGVI Score at Year End:** | | **0** | **1** | **2** | **3** | **4** | **5** |
| **Freedom from Aortic Complications** | **Low-risk** | - | 99% | 99% | 96% | 91% | 88% |
|  | **High-Risk** | - | 97% | 95% | 90% | 70% | 55% |
| **Number at Risk** | **Low-risk** | 220 | 191 | 141 | 91 | 49 | 21 |
|  | **High-Risk** | 66 | 57 | 43 | 28 | 13 | 4 |

**Appendix D: Simulated Surveillance Protocols**

To compare the performance of ANN versus other methods, such as the maximum diameter of the AAA prior to EVAR, a simulation study was conducted.

The “SGVI score” (St George’s Vascular Institute) is a previously validated model for stratifying the risk of EVAR complications, and utilizes the AAA and CIA diameter prior to EVAR to allocate patients to a high-risk or low-risk group[^17^](#_ENREF_17). The SGVI Score is calculated as (0.03675 * maximum AAA diameter) + (0.05009 * largest common iliac diameter), and a threshold of 3.76571 is used to distinguish between high-risk and low-risk groups[^17^](#_ENREF_17). Simulation studies were conducted to investigate the comparative performance of protocols for risk-stratified surveillance based on the pre-operative SGVI score compared to protocols for risk-stratified surveillance based on the classification of patients using the ANN model. A standard technique was used to generate simulations, based on the predicted survival curve of every patient based on a Cox Proportional Hazards model[^45^](#_ENREF_45); a Weibull distribution was fitted to summarise the survival curve of each patient, and used to generate 1000 simulated times to endograft complication. In each case of a simulated complication, a maximum sojourn time of 133.98 days was applied; a complication was assumed to develop with equal probability throughout this interval, and randomly generated. In cases where a surveillance scan occurred between the simulated onset of a complication and the simulated onset of symptoms, the patient was considered detected by the surveillance. A risk-stratified surveillance protocol for the high-risk and low-risk group has been defined previously[^17^](#_ENREF_17) and was applied to the present study population. Event detection rates were compared to a standard surveillance protocol from our institution (9 scans in 5 years). A cost-neutral alternative risk-stratified protocol was tested, which allocated high-risk patients to 12 simulated scans in 5 years (at 90, 180, 270, 365, 480, 600, 730, 870,1095, 1275, 1460 and 1650 days) and low-risk patients to 8 simulated scans in 5 years (at 90, 270, 450, 750, 1000, 1250, 1680 and 1770 days).

Comparisons of simulated surveillance demonstrated that ANN-based surveillance resulted in significantly greater discrimation of event detected rates compared to SGVI score-based surveillance:


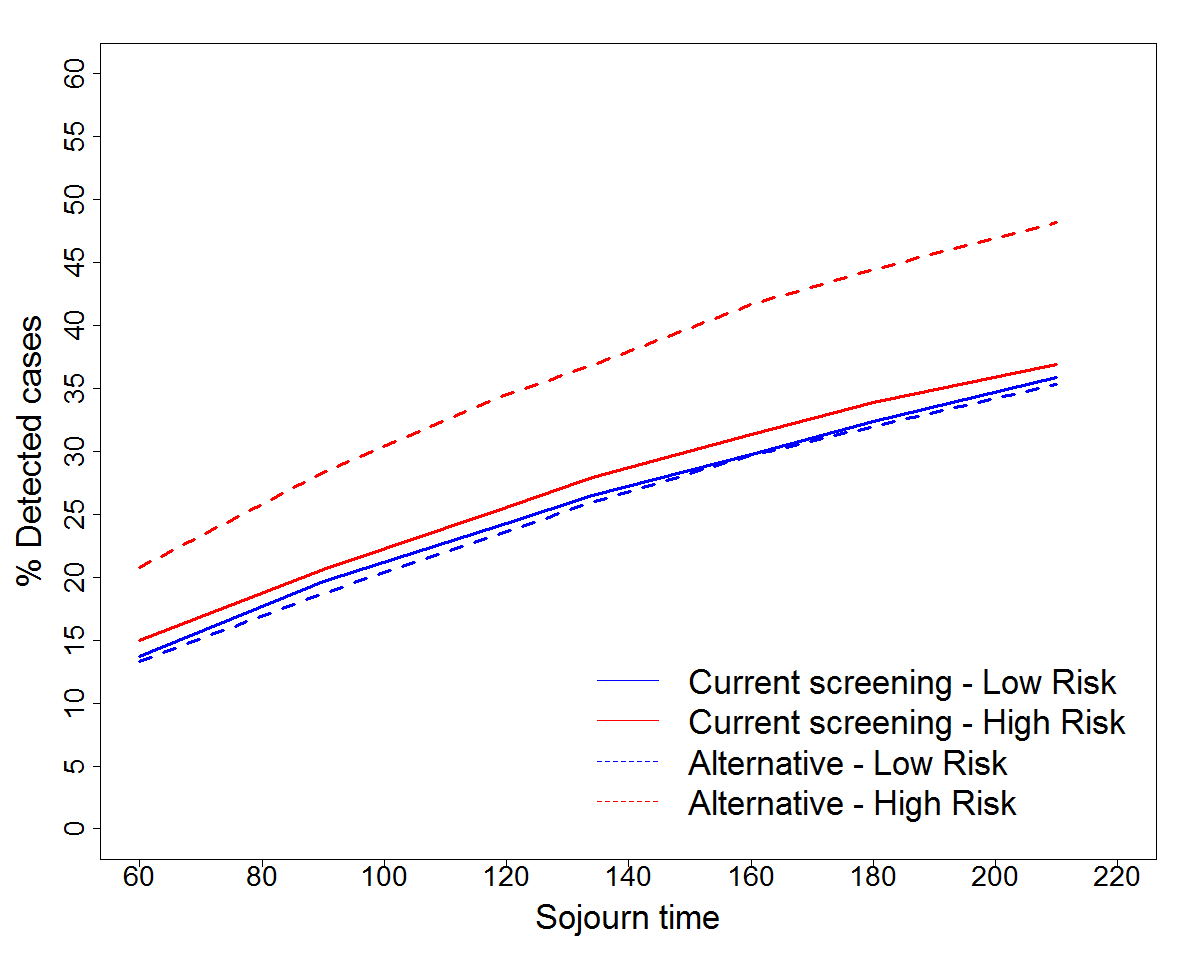


*Appendix D, Figure 1. Simulation Study of Risk-Stratified Surveillance after classification of patients using the existing SGVI score (Current Screening) compared to classification of patients using the ANN model (Alternative).*

**Appendix E: Performance of a 3-group ANN for classification of high-risk, medium-risk, and low-risk patients for limb or aortic endograft complications after EVAR.**


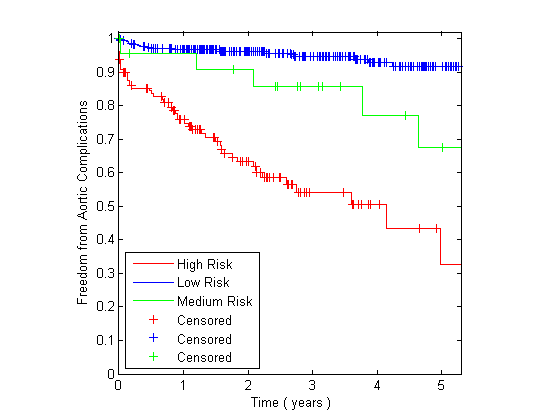
**Appendix E, Figure 1: Performance of a 3-group ANN for classification of high-risk, medium-risk and low-risk patients for limb or aortic endograft complications after EVAR in centre 1:**

| **Year** | | **0** | **1** | **2** | **3** | **4** | **5** |
| --- | --- | --- | --- | --- | --- | --- | --- |
| **Freedom from Limb and Aortic Complications** | **Low-risk** | - | 96.6% | 96.2% | 94.5% | 92.7% | 91.7% |
|  | **Medium-Risk** | - | 90% | 85.6% | 85.6% | 77% | 67% |
|  | **High-Risk** | - | 75.8% | 63% | 54% | 43% | 32.5% |
| **Number at Risk** | **Low-risk** | 310 | 265 | 203 | 149 | 91 | 44 |
|  | **Medium-risk** | 23 | 20 | 18 | 13 | 9 | 6 |
|  | **High-Risk** | 131 | 80 | 44 | 17 | 8 | 3 |

| **Risk Group** | **p-value** |
| --- | --- |
| **Low vs. Medium risk** | 0.02249 |
| **Low vs. High-risk** | ‹ 0.00001 |
| **High-risk vs. Medium risk** | 0.01877 |

**Appendix E, Figure 2: Performance of a 3-group ANN for classification of high-risk, medium-risk and low-risk patients for limb or aortic endograft complications after EVAR in centre 2:**

**N=8 (number of hidden neurons)**

| **Year** | | **0** | **1** | **2** | **3** | **4** | **5** |
| --- | --- | --- | --- | --- | --- | --- | --- |
| **Freedom from Limb and Aortic Complications** | **Low-risk** | - | 97% | 97% | 94.6% | 94.6% | 94.6% |
|  | **Medium-Risk** | - | 96% | 96% | 91.6% | 91.6% | 91.6% |
|  | **High-Risk** | - | 90% | 89% | 85% | 82.7% | 82.7% |
| **Number at Risk** | **Low-risk** | 141 | 130 | 98 | 64 | 30 | 12 |
|  | **Medium-risk** | 50 | 44 | 30 | 19 | 13 | 2 |
|  | **High-Risk** | 95 | 74 | 56 | 35 | 19 | 3 |

**
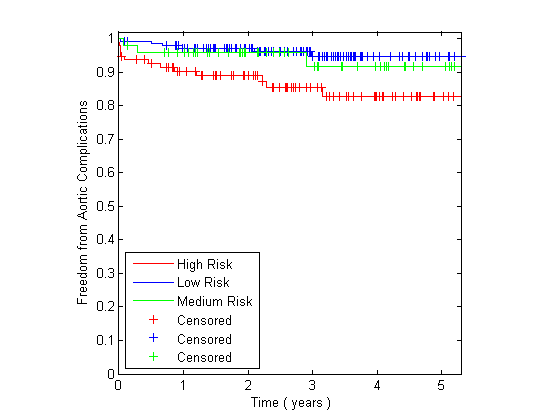
**

| **Risk Group** | **p-value** |
| --- | --- |
| **Low vs. Medium risk** | 0.88470 |
| **Low vs. High-risk** | 0.01305 |
| **High-risk vs. Medium risk** | 0.21799 |
